# Post-Traumatic Growth and Quality of Life Among World Trade Center Health Registry Enrollees 16 Years After 9/11 †

**DOI:** 10.3390/ijerph23030393

**Published:** 2026-03-20

**Authors:** Howard E. Alper, Leen Feliciano, Lucie Millien, Cristina Pollari, Sean Locke

**Affiliations:** 1 World Trade Center Health Registry, New York City Department of Health and Mental Hygiene, Division of Epidemiology, New York, NY 11101, USA; lmillien@health.nyc.gov (L.M.); cdpollari@gmail.com (C.P.); seanhlocke@gmail.com (S.L.); 2 Graduate School of Public Health and Health Policy, The City University of New York, New York, NY 10027, USA; leen.feliciano43@sphmail.cuny.edu

**Keywords:** 9/11, post-traumatic growth, World Trade Center, quality of life, SF-12, physical function, mental function

## Abstract

**Highlights:**

**Public health relevance—How does this work relate to a public health issue?**

- The prevalence of Post-Traumatic Growth in the World Trade Center Health Registry was 31%.
- Post-Traumatic Growth predicted mental but not physical Quality of Life.

**Public health significance—Why is this work of significance to public health?**

- What is the prevalence of Post-Traumatic Growth in the World Trade Center Health Registry? The prevalence of Post-Traumatic Growth in the World Trade Center Health Registry is 31%.
- Does Post-Traumatic Growth predict Quality of Life? Post-Traumatic Growth predicts mental but not physical Quality of Life.

**Public health implications—What are the key implications or messages for practitioners, policy makers and/or researchers in public health?**

- Post-Traumatic Growth is common after a disaster and is associated with improved mental Quality of Life.
- Researchers should explore interventions to augment Post-Traumatic Growth.

**Abstract:**

A recent study of World Trade Center Health Registry enrollees found that about one- third experienced post-traumatic growth (PTG) in the wake of the 9/11 attacks and that PTG was associated with social support and social integration. However, the implications of PTG for the enrollees’ overall quality of life are unknown. The present study investigated the prevalence of PTG and its association with the SF-12 physical and mental function quality of life scales in a sample of 2786 enrollees from the Registry’s Health and Quality of Life Study (HQoL) who completed the first four surveys, were older than 18 on 9/11, reported English as their primary spoken language, and provided consistent self-report of 9/11 physical injury at the Registry’s baseline and HQoL surveys. We employed multivariable linear regression to evaluate the association between PTG and the SF-12 physical and mental scales, controlling for sex, age, race, education, income, employment, social support, threatening events, post-9/11 mental health, number of post-9/11 physical health conditions, and drug/alcohol misuse. We found that 31% of the sample enrollees experienced PTG and that PTG exhibited a clinically and statistically significant association with the SF-12 mental scale but not the physical scale (physical: β = −0.01 (−0.61, 0.65), mental: β = 3.92 (2.89, 4.95)). Those who were physically injured during 9/11 showed larger improvements in mental function than those who were not. PTG has implications for the overall mental quality of life that should be further investigated.

## 1. Introduction

The World Trade Center (WTC) terrorist attacks on 11 September 2001 resulted in over 2700 deaths and many thousands more physically injured. The dust/debris cloud generated by the collapse of the WTC towers enveloped many survivors and responders, who also may have been physically injured or witnessed horrific events such as seeing airplanes strike the towers. These exposures to the 9/11 attacks have been found to be associated with a variety of physical and mental conditions, such as asthma [1,2,3,4,5], post-traumatic stress disorder (PTSD) [6,7,8,9], heart disease [6,10], stroke [8], cancer [11,12], autoimmune disease [13], and somatic symptoms [14].

Although research by the Registry and others has shown that exposure to traumatic events can have deleterious implications, recent studies have discovered significant differences in how individuals respond to trauma [15,16]. Specifically, some individuals report marked improvements in their quality of life (physical and mental function) following a traumatic event [17,18,19]. The positive psychological changes that occur in response to the traumatic event may result in higher levels of functioning, meaning that the individuals do not return to their pre-trauma levels of functioning [20,21].

This phenomenon, known as post-traumatic growth (PTG), was originally measured using a scale developed by Tedeschi and Calhoun and has gained widespread attention in public health research [22,23]. PTG can include a positive shift in mindset and beliefs, greater life satisfaction, acceptance of the traumatic event, and improved interpersonal relationships. Individuals who report PTG may also engage in new or changed spiritual beliefs and assign positive meaning to their traumatic experiences.

Recent research has shown that 34.3% of World Trade Center Health Registry (WTCHR) enrollees demonstrated moderate-to-high PTG in the aftermath of direct exposure to the 9/11 attacks [24]. Exploring the association between PTG and quality of life is useful to understand the long-term physical and mental health effects of 9/11 and to inform new policies and treatments. Further, it is important to investigate this association separately for those with and without physical injuries on 9/11, because injury on 9/11 has been shown to be associated with the subsequent development of PTSD [25] and heart attack. Diseases such as these could affect both PTG and quality of life and, therefore, their association.

The mechanism underlying the relationship between PTG and health-related quality of life (HRQOL) is not well-understood. Several systematic reviews have evaluated PTG and HRQOL but have found conflicting results. One review did not find a statistically significant association between PTG and the physical functioning domain of HRQOL [26]. Another review found that half the studies included revealed no relationship between PTG and HRQOL, while others found a positive relationship [27]. A recent review from 2020 found a positive relationship between PTG and HRQOL among cancer patients, indicating that PTG may play an integral part in successful coping [28]. More research is needed to gain insight into the complex relationship between PTG and HRQOL. Further, other mental health conditions, such as depression, could affect the association between PTG and HRQOL. The present study tests the hypothesis that PTG is positively associated with HRQOL. The aims of this study were to (1) determine the prevalence of post-traumatic growth (PTG) among World Trade Center Health Registry (WTCHR) enrollees with direct exposure to 9/11, (2) evaluate the association between PTG and physical and mental function in a sample of physically injured plus non-injured enrollees, and (3) evaluate this association separately for physically injured and non-injured enrollees. The present study is a reanalysis of a previously published paper on this topic [29].

## 2. Materials and Methods

### 2.1. Participants

The New York City Department of Health and Mental Hygiene (NYC DOHMH) established the WTCHR in 2002 to monitor the long-term physical and mental health effects of the attacks on 11 September 2001 (9/11). The Registry conducted its initial survey in 2003–2004 (Wave 1), which included rescue and recovery workers, area residents, area workers, passersby, and students and staff in local schools. An additional three health surveys have been conducted: Wave 2 in 2006–2007, Wave 3 in 2011–2012, and Wave 4 in 2015–2016. Details describing the Registry’s methods for recruitment and data collection are available in previous publications [3,30].

In 2017–2018, the Registry conducted the Health and Quality of Life (HQoL) survey [31], to investigate the potential association between physical injury on 9/11 and quality of life, using measures for the latter such as the SF-12 overall physical and mental function [21], the SSS-8 somatic symptoms scale, the GRIT scale, Post-Traumatic Growth Inventory [22,23], etc. Participation was limited to enrollees who had completed all four Registry survey waves fielded to date, were at least 18 years of age at Wave 1, whose primary spoken language was English, and who were located below Chambers Street on 9/11. The Registry consists of 71,423 enrollees, and approximately 35,000 enrollees were excluded due to the above criteria. Further, two groups were included in the survey. The first group consisted of all enrollees who reported having one or more of the following 9/11-related physical injuries on the Wave 1 survey: cut, abrasion, or puncture wound; sprain or strain; burn; broken bone or dislocation; and concussion or head injury. Those who reported “other injury” or “eye injury” were excluded. The second group consisted of a random selection of enrollees from the Wave 1 survey who reported no physical injury during Wave 1. This second group was chosen to be similar in size to the first, to keep the total HQoL sample relatively small compared with the full Registry. Furthermore, for the present study, only enrollees who reported consistent responses for sustaining a 9/11-related physical injury on both Wave 1 and the injury and HQoL survey were included in the current analysis (e.g., “yes” on Wave 1 and “yes” on the injury and HQoL survey or “no” on both). Those who did not have complete data on the exposures, outcome, and covariates were excluded. No significant differences were observed between those who were included and excluded from the study, in that all injured enrollees from the total Registry population were included in the study sample, and non-injured enrollees in the study sample were a random sample of all the non-injured enrollees in the Registry. The Institutional Review Boards at the Centers for Disease Control and Prevention and the NYC DOHMH approved the Registry’s protocol and use of data. Further details regarding the creation of the HQoL survey including recruitment and outcomes are available in a prior Registry publication [30].

### 2.2. Outcome

The HQoL survey included a series of 12 questions known as the Short-Form Health Survey-12, Version 1 (SF-12) [21]. The SF-12 was derived from the SF-36 to provide an efficient method for assessing overall physical and mental health functioning through a mean physical health component summary score (PCS-12) and a mean mental health component summary score (MCS-12). The summary scores are based on combinations of SF-12 questions that were identified as representing the overall physical and mental status and that were highly correlated with the concomitant SF-36 scores. This is described in detail in a previous publication [32]. For both the physical and mental summary scores, higher values corresponded to better, healthier functioning.

### 2.3. Exposure

Post-traumatic growth was evaluated using the Post-Traumatic Growth Inventory (PTGI), a self-report 21-item scale developed by Tedeschi and Calhoun to assess five domains of positive change following trauma: new possibilities, relating to others, personal strength, spiritual change, and appreciation of life [22,23]. Participants specified their level of agreement with each item on the PTGI, which involved statements about positive change experienced after trauma, such as “I have a greater appreciation for the value of my own life” and “I have more compassion for others”. PTGI Item 7, “I established a new path for my life”, was unintentionally omitted from the HQoL survey, so it was not distributed to participants. As a result, PTGI Item 7 was excluded from all analyses. A total PTGI score was calculated from the sum of the remaining 20 PTGI items. The PTGI uses a 5-point Likert scale to rank responses from 0 (“I did not experience this change as a result of my crisis”) to 5 (“I experienced this change to a very great degree as a result of my crisis”). Therefore, the total PTGI score ranged from 0 to 100. Average PTGI scores (total/20) ≥ 3 were taken as indicative of moderate-to-high post-traumatic growth.

### 2.4. Covariates

Demographic and risk factors were obtained from the Registry’s wave datasets: wave 1 (2003–2004), wave 2 (2006–2007), wave 3 (2011–2012), and wave 4 (2015–2016).

The demographic characteristics included in the current analysis include sex (male, female), age at 9/11, race (White Non-Hispanic, Black Non-Hispanic, Hispanic, Asian, or Multiracial/other), education (less than high school, high school/GED, some college, or college/post-grad), and income (0 ≤ USD 25 K, USD 25 ≤ USD 50 K, USD 50 ≤ USD 75 K, USD 75 ≤ USD 150 K, ≥ USD 150 K), and eligibility group (rescue/recover worker, resident, area worker, passerby, or student/staff).

Risk factors included employment status, social support, life-threatening events post- 9/11, mental health conditions post-9/11, physical health conditions post-9/11, and alcohol or drug use post-9/11. Employment status was defined as “yes” if participants were employed full-time/part-time/self-employed during Wave 4 and as “no” if the enrollee reported otherwise. Information on social support was collected during Wave 3. Research has shown that little to no social support results in decreased mental function and worsens the severity of pre-existing mental health conditions such as PTSD, depression, and possibly somatic symptom disorder [14]. Participants were asked the following five questions to measure social support: “How often is someone available to take you to the doctor if you need to go?”, “to have a good time with you?”, “to hug you?”, “to prepare your meals if you are unable to do it yourself?”, and “to understand your problems?”. Responses were rated from 0 (“none of the time”) to 4 (“all of the time”). A total score for social support consisted of the sum of all five questions and ranged from 0 to 20, with low social support defined as a total score below 15. Similarly, exposure to life-threatening events can result in decreased mental function and add to mental health issues such as PTSD and depression [33]. Participants either confirmed or denied up to eight life-threatening events post-9/11 (such as having experienced a disaster, accident, or threat with a weapon) during Wave 3. The number of life-threatening events experienced was based on the total number of affirmative responses and expressed categorically as none (0 events), low/medium (1 or 2 events), and high (greater than 2 events). Mental health post-9/11 was evaluated by asking participants if they experienced PTSD, anxiety, or depression between the baseline and Wave 4 surveys. Physical health post-9/11 was categorically expressed as having none, one, or two or more of the following chronic diseases: asthma, hypertension, angina, heart attack, stroke, diabetes, coronary heart disease, gastroesophageal reflux disease, rheumatoid arthritis, multiple sclerosis, or peripheral neuropathy, between the baseline and Wave 4 surveys. Research has shown that alcohol and drug misuse lead to poor physical and mental health status and may worsen pre-existing PTSD and depression [34,35,36,37]. Participants were asked the following question during Wave 4 to determine the problem of alcohol or drug use post-9/11: “Have you ever stayed overnight or longer at a hospital, rehabilitation facility, or mental health center so you could receive treatment or counseling for alcohol or drug use?” Participants either confirmed or denied a problem with alcohol and drug misuse and specified if this occurred before 9/11, after 9/11, or both before and after 9/11. Those who only reported alcohol and drug use before 9/11 were excluded from the current analysis.

### 2.5. Statistical Analysis

The primary outcomes of this analysis were the physical and mental health summary component scores (PCS-12 and MCS-12) of the SF-12. The mean PCS-12 and MCS-12 scores were evaluated for PTG, plus sociodemographic variables and risk factors, for the entire analytic sample and separately by injury status. The distributions for the above categorical variables were also calculated.

A bivariate analysis was conducted for the entire analytic sample to evaluate the association between PTG and physical and mental function.

The associations between PTG and overall physical and mental function were obtained from multivariable linear regressions of PCS-12 and MCS-12 on PTG, controlling for the above sociodemographic and risk factors (including rescue/recovery worker status). While the main analysis concerned the region below Chambers Street, we performed similar regressions for the region between Canal and Chamber Streets for purposes of comparison. The resulting regression coefficients represented the extent of change in the PCS-12 or MCS-12 score relative to the reference category for the variable of interest. *p*-values and 95% confidence intervals for the betas were also calculated. The significance level was set at a 2-sided value of alpha < 0.05. All analyses were conducted using SAS 9.4 (SAS Institute, Inc., Cary, NC, USA).

## 3. Results

### 3.1. Demographic Characteristics of the Total Sample

The study sample consisted of 2786 enrollees, 985 who were injured on 9/11 and 1801 who were not injured. Most participants were White Non- Hispanic (75%), male (56%), and aged 25–44 at 9/11 (51%). Approximately 64% of participants completed a bachelor’s degree or post-graduate education, and 63% reported employment during Wave 4 (2015–2016), with 40% reporting an average annual income greater than greater than USD 75,000 USD but less than USD 150,000 USD during Wave 1 (Table 1).

More than half of the participants reported moderate-to-high levels of social support (55%) and a low number of threatening events (69%) at Wave 3. Most participants reported no post-9/11 mental disease (67%) and no post-9/11 problem with alcohol or drugs (96%), and a minority reported no post-9/11 physical disease (43%) (Table 1).

For the present study sample, Cronbach’s α = 0.96 for the PTGI, α = 0.82 and α = 0.89 for the SF-12 physical and mental scales.

### 3.2. Prevalence of PTG

The overall prevalence of PTG among WTCHR Enrollees in the sample was 31% (Table 1).

### 3.3. The Bivariate Associations of PTG with SF12

Participants with moderate-to-high PTG were more likely to show improved mental function in the bivariate analysis (β = 3.50, 95% CI: 2.47, 4.52; *p* ≤ 0.05) (Table 2). There was a small decline in physical functioning (β = −0.77, 95% CI: −1.38, −0.15; *p* ≤ 0.05).

### 3.4. Multivariate Associations of PTG with SF12

Previous research on the minimal clinically important difference between PCS-12 and MCS-12 in patients [38] revealed β ≥ 3.29 for both physical and mental function as clinically meaningful. Specifically, such changes in PCS12 have been associated with meaningful differences in functional mobility, regular daily activities, and bodily pain. For MCS12, changes ≥ 3.29 have been associated with meaningful differences in psychological distress and social functioning. Participants with moderate-to-high PTG were more likely to show clinically significant improvements in mental function in the multivariate analysis (β = 3.92, 95% CI: 2.89, 4.95; *p* ≤ 0.05). The association between PTG and mental function was enhanced in those participants who also experienced one or more physical injuries during 9/11 (β = 4.98, 95% CI: 3.19, 6.77; *p* ≤ 0.05), meaning that injured enrollees mental function improved clinically more than for the entire sample. There was no association between PTG and physical functioning for the sample, the physically injured group, or the physically non-injured group (Table 2), implying that PTG did not result in clinical improvements in physical functioning for our study sample.

## 4. Discussion

This study evaluated the prevalence of post-traumatic growth and its association with overall Health and Quality of Life (HQoL). We found that 31% of the sample demonstrated PTG. In addition, PTG was found to be clinically and substantially related to mental function but not to physical functioning. The positive association observed between PTG and mental function supports the notion that PTG is a psychological process born out of a careful examination and positive interpretation of traumatic events [23,39,40]. This is also consistent with other studies that show an association between PTG and improved mental function in military personnel [41,42,43].

In terms of gender, other studies have reported lower MCS-12 and PCS-12 scores for women when measuring health-related quality of life using the SF-12 [44,45]. The differences observed for age are consistent with the literature on the SF-12 showing that mental health tends to improve with age, while physical health declines [46,47,48]. The role of education in promoting physical health and functioning has been well-documented in the literature [49,50,51]. The increased physical and mental function observed in conjunction with increases in annual income are to be expected given that income improves access to health care [52]. Our findings on social support and quality of life are consistent with the findings of other studies that link higher levels of social support to better physical and mental health outcomes [53,54,55]. Our finding that physical and mental health decline with increased traumatic events or threats is consistent with that reported in the literature [56,57,58]. The enhanced associations of PTG with both physical and mental function for physically injured enrollees may result from the increased adversity arising from physical injury, compared with non-injury.

Our results for the association of PTG with the SF-12 are consistent with the results of other investigations into the association of PTG with quality of life (QoL). For example, a study of North Korean defectors to South Korea [59] found that PTG was positively associated with a World Health Organization (WHO) QoL measure for quality of life. Another study, of the 2011 Tohoku earthquake in Japan [60], performed trajectory analyses of PTG and found three trajectories: no growth, illusory growth, and growth. QoL was seen to be positively associated with the probability of being in this sequence of PTG trajectories. Finally, another study of traumatized psychiatric patients [18] found that PTG was associated with QoL and that PTG explained more of the variance in the QoL outcomes than PTSD or any other variable in the model.

PTG has received much attention in disaster and other types of studies in the literature in recent years. However, there are conceptual and methodological issues with PTG. It only measures positive change and does not account for post-traumatic decline or stasis. Further, doubts persist as to whether self-reported PTG represents “true” post-traumatic growth or an illusion of growth grounded in coping strategies. One way to attempt to answer this latter question is to determine if self-reported PTG predicts outcomes of interest (e.g., QoL, self-reported chronic disease, hospitalizations). This study provides provisional answers to the above concerns, in that PTG was found to be associated with the SF-12 mental health but not the physical health measure of overall functioning. Future research will investigate the PTG–SF-12 association longitudinally, as well as the predictive validity of PTG by examining the association of PTG with self-report for mental health diseases such as PTSD, depression, and psychological distress.

### Strengths and Limitations

A strength of this study is the use of a prospective cohort, which allowed us to investigate the association between exposures to, and sequelae of exposure to, the 9/11 attacks and later emerging conditions. We also had a sufficiently large sample size to ensure adequate power to detect the PTG–SF-12 association. Further, the sample was composed of physically injured and non-injured enrollees, which allowed the separate determination of the PTG–SF-12 association for these groups. The present investigation also used a well-validated measure of physical and mental function.

One limitation is that though the Registry is a cohort, the HQoL study was cross-sectional, so the directionality of the PTG-PCS12/MCS12 association could not be evaluated. A second limitation is that this study included only enrollees whose primary spoken language was English, which introduced a bias in our ability to generalize our results to the entire Registry population. Another limitation was that data for PTG and covariates were self-reported. However, there has been good agreement between Registry findings based on self-report and those based on hospitalization data, at least for exposure–disease associations [61]. Additionally, the HQoL sample is a non-random sample of the Registry population, so inferences from this study may not be generalized to the latter. Finally, the PTGI is self-reported, and previous research has shown only modest agreement with either more objective behavior measures or reports from the friends of the subjects [62,63].

## 5. Conclusions

We investigated the association between post-traumatic growth and overall physical and mental function in a sample of World Trade Center Health Registry enrollees. We found that post-traumatic growth was associated with the overall mental function, but not physical functioning, in WTCHR enrollees 16 years post-9/11. Specifically, moderate-to-high PTG was found to be clinically and substantially related to mental function. Participants who experienced physical injuries during 9/11 showed greater levels of moderate-to-high PTG and a stronger association of PTG with overall mental function.

## Figures and Tables

**Table 1:** Exposure and sociodemographic frequency distributions and physical and mental SF-12 scores.

	**Full Sample**				**Injured Only**				**Non-Injured Only**			
Variable	N	%	PCS-12 (Mean)	MCS-12 (Mean)	N	%	PCS-12 (Mean)	MCS-12 (Mean)	N	%	PCS-12 (Mean)	MCS-12 (Mean)
Post-Traumatic Growth												
Yes	844	30.84	39.75	49.68	351	36.49	38.04	46.84	493	27.77	40.91	51.62
No	1893	69.16	40.65	46.15	611	63.51	37.28	40.99	1282	72.23	42.15	48.46
Sex												
Male	1564	56.14	40.24	48.54	602	61.12	37.14	44.59	962	53.41	42.13	50.95
Female	1222	43.86	40.56	45.44	383	38.88	38.29	40.44	839	46.59	41.46	47.42
Age at 9/11												
0–17	13	0.47	50.25	32.13	1	0.10	50.25	32.13	12	0.67	50.25	32.13
18–24	124	4.45	44.94	46.51	28	2.84	43.06	41.44	96	5.33	45.37	47.68
25–44	1415	50.79	40.95	45.89	521	52.89	37.99	42.26	894	49.64	42.60	47.90
45–64	1208	43.36	39.29	48.96	427	43.35	36.78	44.27	781	43.36	40.55	51.31
65 or older	26	0.93	32.06	50.22	8	0.81	27.44	45.34	18	1.00	33.32	51.55
Race/Ethnicity												
White non-Hispanic	2080	74.66	40.64	47.88	705	71.57	37.65	44.10	1375	76.35	42.12	49.75
Black non-Hispanic	263	9.44	40.08	45.04	110	11.17	38.71	37.54	153	8.50	40.93	49.68
Hispanic	263	9.44	38.68	43.70	114	11.57	36.53	40.79	149	8.27	39.99	45.48
Asian	105	3.77	39.93	47.40	30	3.05	36.13	44.56	75	4.16	41.35	48.46
Other	75	2.69	40.52	47.65	26	2.64	35.72	44.19	49	2.72	43.00	49.44
Education												
High School/GED	413	14.85	38.23	45.83	207	21.06	36.26	41.71	206	11.46	39.80	49.11
Some College	577	20.75	38.52	45.86	284	28.89	36.70	42.49	293	16.30	40.29	49.14
College/Post Grad	1791	64.40	41.44	47.95	492	50.05	38.54	43.97	1299	72.25	42.50	49.38
Income at Wave 1												
<$25 K	153	6.05	40.36	40.64	75	8.35	36.38	35.52	78	4.78	43.29	44.42
$25–<50 K	387	15.30	40.19	44.30	165	18.37	37.95	41.15	222	13.61	41.64	46.34
$50–<75 K	533	21.08	39.47	46.10	192	21.38	36.65	42.12	341	20.91	40.96	48.20
$75–<150 K	1019	40.29	40.00	48.56	361	40.20	36.94	44.63	658	40.34	41.62	50.64
$150 K or more	437	17.28	42.40	49.42	105	11.69	41.06	45.69	332	20.36	42.83	50.61
Employed at Wave 4												
Yes	1743	62.56	42.00	47.92	511	51.88	39.51	44.25	1232	68.41	42.99	49.38
No	1043	37.44	37.44	45.94	474	48.12	35.23	41.74	569	31.59	39.14	49.16
Social Support Wave 3												
Yes	1510	55.17	41.04	50.35	455	47.45	38.17	47.29	1055	59.34	42.26	51.66
No	1227	44.83	39.55	43.29	504	52.55	36.96	39.06	723	40.66	41.19	45.96
Threatening Events, Wave 3												
Low	1916	69.29	41.21	48.26	524	53.85	38.54	43.92	1392	77.68	42.13	49.77
Medium	464	16.78	39.82	45.32	185	19.01	37.42	41.23	279	15.57	41.25	47.75
High	185	6.69	37.98	44.43	109	11.20	36.95	42.63	76	4.24	39.47	47.07
Very High	200	7.23	36.08	44.40	155	15.93	35.04	43.02	45	2.51	39.71	49.24
Post 9/11 Mental Condition												
Yes	918	32.95	40.88	50.82	519	52.69	37.36	38.07	399	22.15	41.67	41.69
No	1868	67.05	39.33	39.72	466	47.31	37.77	48.61	1402	77.85	41.86	51.25
Post 9/11 Physical Condition												
0	1209	43.40	42.85	49.76	251	25.48	41.39	45.83	958	53.19	43.20	50.71
1	808	29.00	40.57	46.76	287	29.14	38.52	43.45	521	28.93	41.62	48.45
2 or more	769	27.60	36.39	43.74	447	45.38	34.95	41.44	322	17.88	38.22	46.66
Post 9/11 Alcohol/Drugs												
Yes	92	3.75	38.95	39.22	50	5.92	37.01	35.76	42	2.61	41.26	43.33
No	2360	96.25	40.43	47.50	795	94.08	37.59	43.54	1565	97.39	41.83	49.46

**Table 2:** Association between post-traumatic growth and HQoL.

	Physical Functioning		Mental Function	
	β	95% CI	β	95% CI
**Sample Between Canal and Chambers Sts**				
Full (injured + non-injured) ^a^	−0.36	(−1.08, 0.37)	4.14	(2.95, 5.32)
Full (injured + non-injured) ^b^	0.17	(−0.56, 0.91)	3.20	(2.06, 4.33)
Injured Only ^b^	−1.50	(−4.35, 1.35)	3.32	(−0.90, 7.54)
Non-Injured Only ^b^	0.25	(−0.50, 1.00)	3.15	(1.97, 4.34)
**Sample Below Chambers St**				
Full (injured + non-injured) ^a^	−0.77	(−1.38, −0.15)	3.50	(2.47, 4.52)
Full (injured + non-injured) ^b^	−0.01	(−0.61, 0.65)	3.92	(2.89, 4.95)
Injured Only ^b^	0.67	(−0.39, 1.73)	4.98	(3.19, 6.77)
Non-Injured Only ^b^	−0.10	(−0.89, 0.69)	3.37	(2.10, 4.65)

^a^—Bivariate regression; ^b^—multivariate regression controlling for sex, age, race and ethnicity, education, income, employment, social support, threatening events, post-9/11 mental health, number of post-9/11 physical health conditions, and drug/alcohol misuse.

## Data Availability

The data presented in this study are available on request from the corresponding author. The data are not publicly available due to subject confidentiality.

## Abbreviations

WTCHR	World Trade Center Health Registry
PTG	Post-Traumatic Growth
CI	Confidence Interval
DSM-IV	Diagnostic and Statistical Manual IV
